# Supplementary material for: Continuous multiplexed population representations of task context in the mouse primary visual cortex
Source: Nat Commun. 2023 Oct 21;14:6687. doi: 10.1038/s41467-023-42441-w (PMC10590415; doi:10.1038/s41467-023-42441-w)
Supplement: Supplementary file 1 — Supplementary Information [file 41467_2023_42441_MOESM1_ESM.pdf]

Supplementary Information

Continuous multiplexed population representations of task context in the mouse primary visual cortex

Márton Albert Hajnal[1,‡], Duy Tran[2,3, ‡], Michael Einstein[2], Mauricio Vallejo Martelo[2], Karen Safaryan[2], Pierre-Olivier Polack[4, †], Peyman Golshani[2,5,6,*,†], Gergő Orbán[1,*,†]

1, Department of Computational Sciences, Wigner Research Center for Physics, Budapest, 1121, Hungary
2, Department of Neurology, David Geffen School of Medicine, University of California, Los Angeles, Los Angeles, CA 90095, United States
3, Albert Einstein College of Medicine, New York, NY 10461, United States
4, Center for Molecular and Behavioral Neuroscience, Rutgers University, Newark, NJ 07102, United States
5, Integrative Center for Learning and Memory, Brain Research Institute, University of California, Los Angeles, Los Angeles, CA 90095, United States
6, West Los Angeles VA Medical Center, CA 90073 Los Angeles, United States
‡, These authors contributed equally
†, These authors jointly supervised this work
*, Corresponding Authors (pgolshani@mednet.ucla.edu, orban.gergo@wigner.mta.hu)

Supplementary Figures 1 − 8

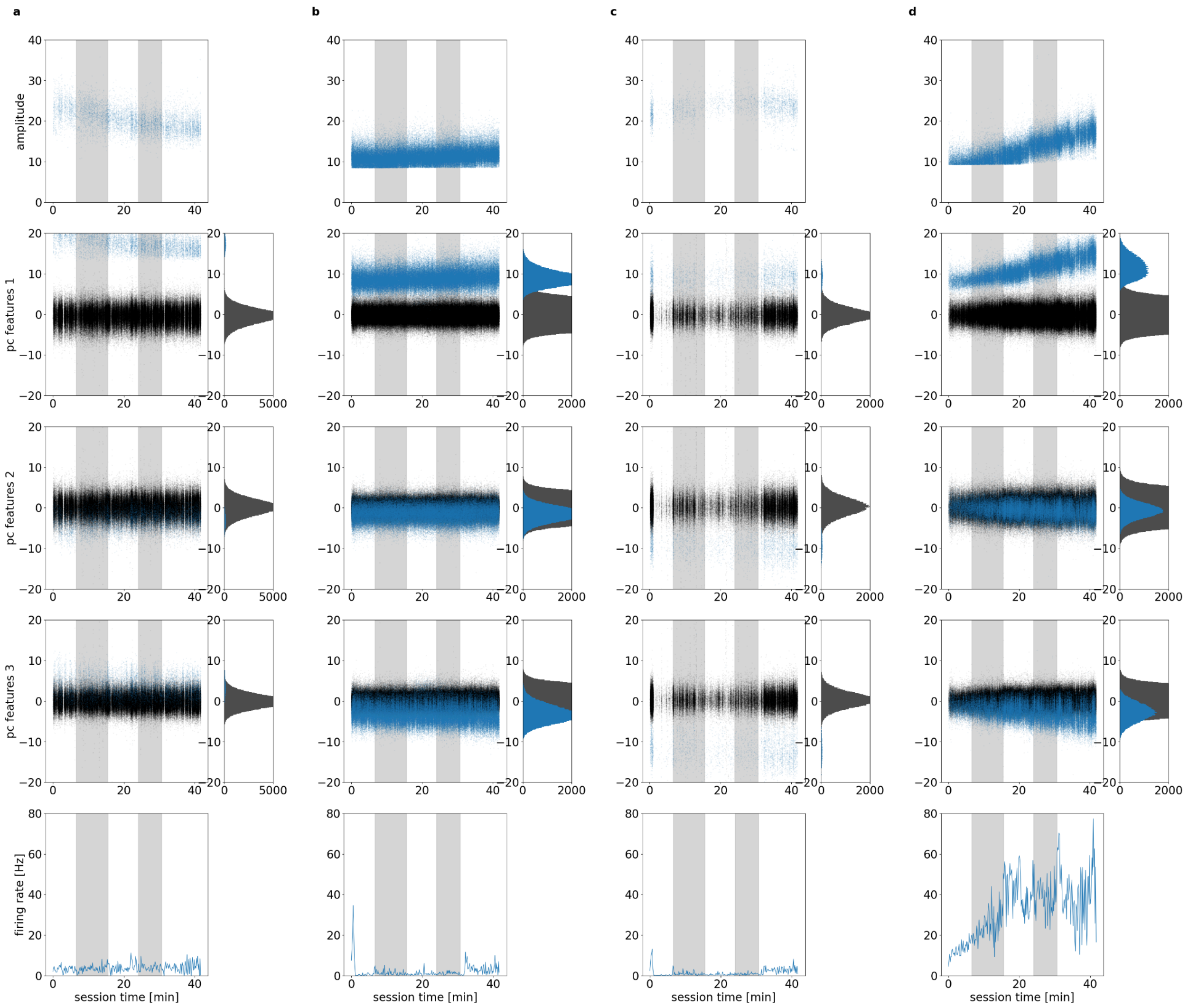

Supplementary Figure 1. Selecting non-drifting units. Example spike characteristics as raw kilosort2 outputs along the recording session (horizontal axis) for trivial and edge case SUAs. *Columns* **a**-**d** are different SUAs. *Top row*: maximum amplitude of waveforms for each spike. *Rows 2,3,4*: wave projections onto the first three PCs of the best channel for the unit (blue dots) and all other units as background noise (black dots); *right side* on each panel: marginal histograms, counted from the total of the whole session in bins width of 0.4 standard principal component units. *Bottom row*: firing rate in 10 seconds bins. Gray shaded areas represent the multimodal stimuli blocks of the first and the second context. **A**, stable SUA, **b**, acceptable non-stationarity above noise ceiling with non-correlating firing rate, **c**, acceptable firing rate, but drifting in the first part of a cluster merge, **d**, visible drift. Drifting SUAs were discarded from further analysis.

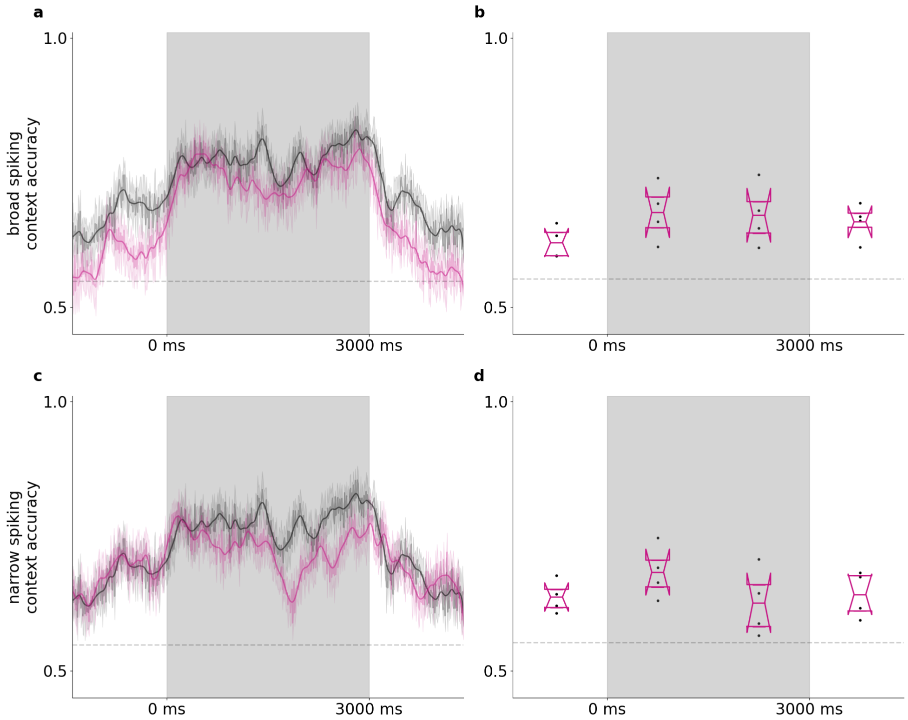

Supplementary Figure 2. Context representation decoded from broad spiking neurons only. **a**, Context decoder accuracy time course using all available units, smoothed from Fig 3E (*black*) and using only broad spiking units (*magenta*) on an example animal. Unsmoothed band is the 2 s.e.m. of cross-validation folds. **b**, Time-averaged mouse-population means of context representation using broad spiking units only, dots are individual mice. **c**, as *a*, but using only narrow spiking units. **d**, as *b*, but using only narrow spiking units. Gray lines are randomized chance levels as in Fig 3. Data from a cohort of 4 animals, where the number of broad and narrow spiking units were >= 20 and >= 10, respectively.

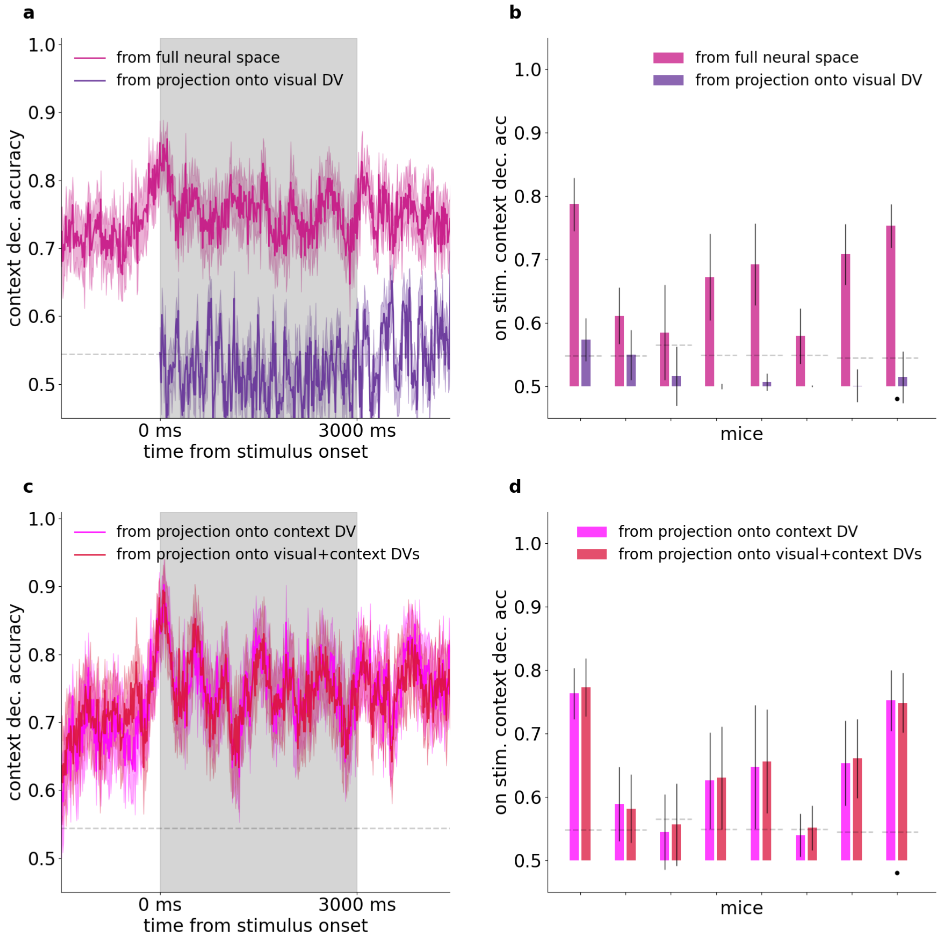

Supplementary Figure 3. Context decoding from neural activity projected onto subspaces formed from combinations of the visual and context decision vector subspaces. **a**, Context accuracy timecourses of an example animal, decoding from the full neural activity space (*magenta*), and from neural activity projected onto the subspace defined by the visual decision vector (*purple*) at each timepoint along the trial. Solid lines are cross-validation averages, with faint bands 2 s.e.m. Gray line is the randomized chance level. **b**, For all mice separately (*bar pairs, horizontal axis*) time-averaged CV-mean accuracies during stimulus presentation, decoding from the full neural activity space (*magenta bars*), and from neural activity projected onto the subspace defined by the visual decision vector (*purple bars*). Error bars represent 1 standard deviation of the distribution of accuracies along the 300 timepoints. Gray lines representing randomized chance level are independently calculated for each mouse. Dot corresponds to the animal on *a*. **c**, context decoder accuracies, as in *a*, but from population activity projected onto the 1D context decision vector with two fold outer crossvalidation (*pink*), and onto a 2D subspace formed from this 1D DV and the 1D visual DV (*red*). **d**, as *b*, but time-averages corresponding to the 1D and 2D subpsaces from *c*, for each mouse.

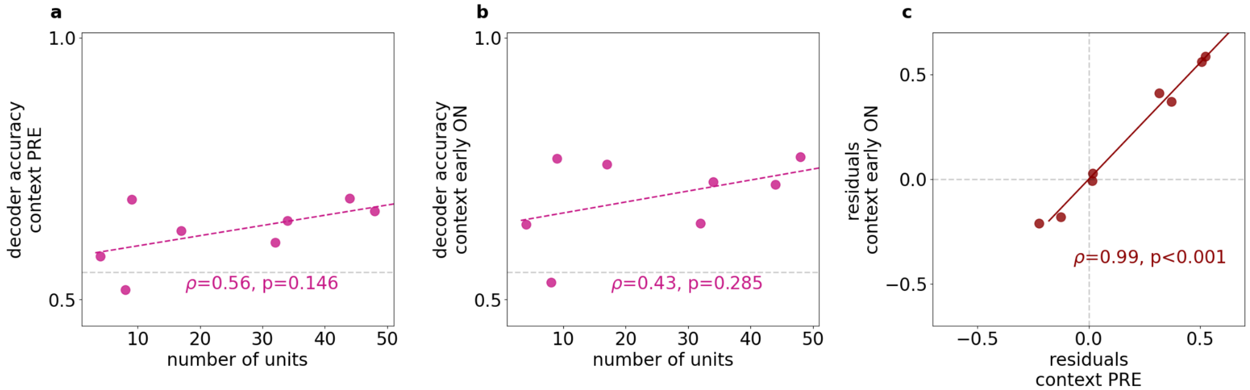

Supplementary Figure 4. Control for number of neurons in context representation. **a**, Accuracy of a decoder cross-validation test accuracy for a task variable is nonsignificantly predicted from the number of units. Dots represent average accuracy of context decoders before stimulus onset for a single animal. Gray lines are animal-averaged randomized chance levels as previously. **b**, same as *a*, but regressed for context average accuracy during stimulus. Gray lines are animal-averaged randomized chance levels as previously. **c**, residuals from the fits from *a* and *b* are correlated, containing no relation between number of neurons and accuracies.

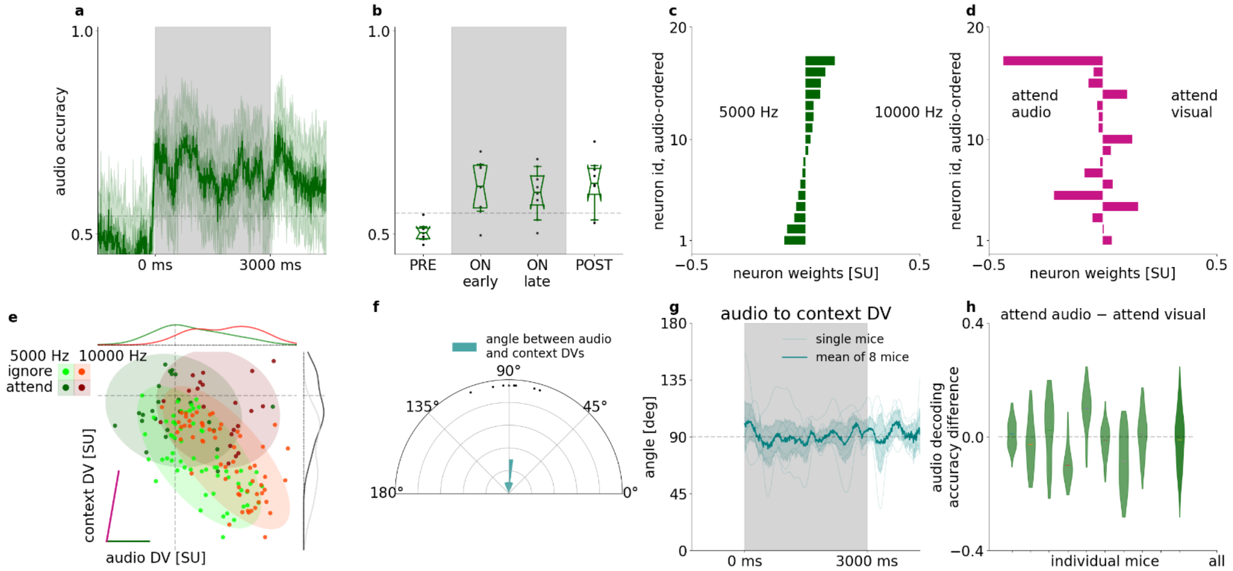

Supplementary Figure 5. Audio variable in V1 is represented orthogonally to context. **a**, Decoder performance in 50 ms time windows at 10 ms sliding resolution for audio stimulus identity for an example animal. Gray shading marks the stimulus presentation period. Gray dashed line indicates shuffled baseline. **b**, Average performance of audio decoder for all animals (*dots*), prior to stimulus onset (PRE), during stimulus (ON), and after stimulus (POST). Box and whiskers denote 25-75, and 2.5-97.5 percentiles respectively, midlines are the mean, notches are 95% confidence level error of the mean. Gray dashed line indicates shuffled baseline averaged over animals. **c**, Contributions of individual neurons (decoder weights) arranged according to the magnitude of the weights. **d**, same *c*, but for context. Ordering of neurons is the same as that on panel *c*. **e**, Population responses averaged over the first 1.5 s of stimulus presentation projected on the DV subspace in individual trials (*dots*) and their estimated normal distribution (mean and 2 std, *shaded ovals*) in different task contexts (*dark* and *light*) and with different audio stimuli presented (*red* and *green*). Purple and green lines denote the DV directions of context and audio decoders, respectively. Histograms show population responses projected on orthogonal components of single DVs. Trials come from multimodal trials in an example animal. **f**, Histogram of the angle between context and audio DVs across animals (*dots*) using average activity in the first 1.5 s of stimulus presentation. **g**, Time course of the angle between the audio and context decoders throughout the trial, distribution of animals. **h**, Distribution (*violins*) with means (*horizontal lines*) of audio decoder accuracy differences between visual and audio context over each trial timepoints for individual mice (left ten plots), and for all mice combined (rightmost)

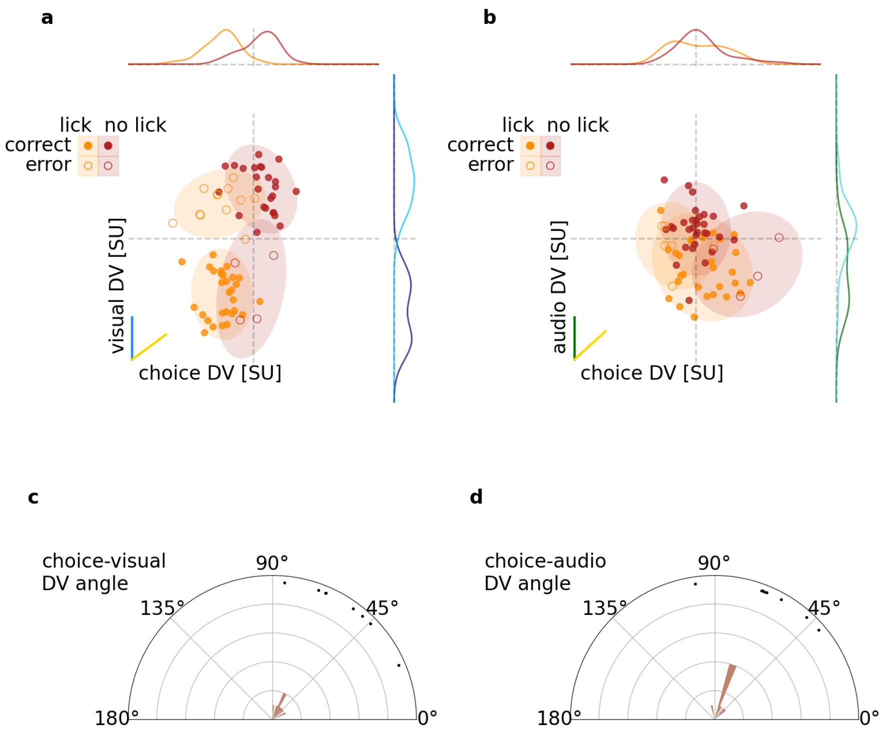

Supplementary Figure 6. Stimulus and choice representations are not orthogonal. **a**, Population responses averaged over the second 1.5 s of stimulus presentation projected on the DV subspace of visual and choice in the attend visual context in individual trials (dots) and their estimated normal distribution (mean and 2 std, *shaded ovals*) for the choices the animals made (red and orange symbols), for an example animal. Open and filled symbols represent if the animal made the correct choice (filled symbols) or committed an error (open symbols). Marginal histograms collect activities projected on the orthogonalized axes. **b**, as *a*, but on the audio and choice subspace in the audio context. **c**, Histogram of the angle between choice and visual DVs across animals (*dots*) using average activity in the first 1.5 s of stimulus presentation. **d**, as in *c*, but between audio and choice in the attend audio context.

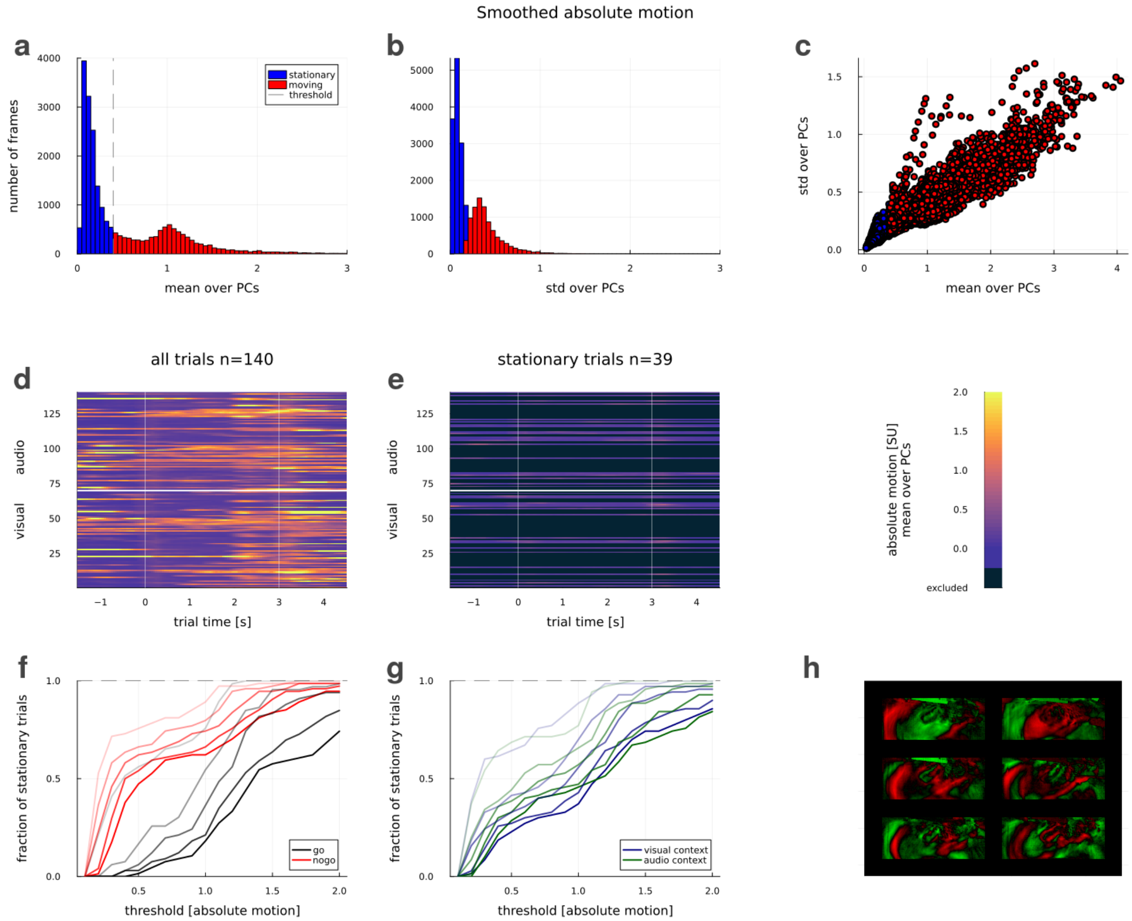

Supplementary Figure 7. Stationary thresholds on video motion PCs. a, Histogram of absolute motion mean over PCs for all time points. Absolute motion below threshold are defined as stationary (*blue*), while above threshold as moving (*red*). **b**, same as *a*, but for the standard deviation (std) over PCs, note that the threshold is based on the mean. **c**, scatterplot of time points, mean versus std. **d**, Absolute motion throughout the time course of a trial (horizontal axis) for each trial (*vertical axis*), in the two contexts (*horizontal white line separator*), color scale (*right*) correspond to panel *a*. **e**, same as *d*, but only stationary trials highlighted, at the threshold chosen in panel *a*, allowing for 10% of timepoints above threshold in each trial. **f**, Fraction of stationary trials at various thresholds (*horizontal axis*) and proportions allowed (*dark to faint lines: 0.05, 0.1, 0.2, 0.3, 0.5*) for go (*black*) and ‘no-go’ (*red*) trials. **g**, Same as *f*, but for trials in visual (*blue*) and audio (*green*) context. **h**, example filters from the differential PCA dimension reduction, that are responsible for various covarying intensity changes: background (*top row*), paw and back (*middle row*), lick, forepaw reach, whiskers, ear (*bottom row*). Red color is decreasing intensity, green color is increasing intensity.

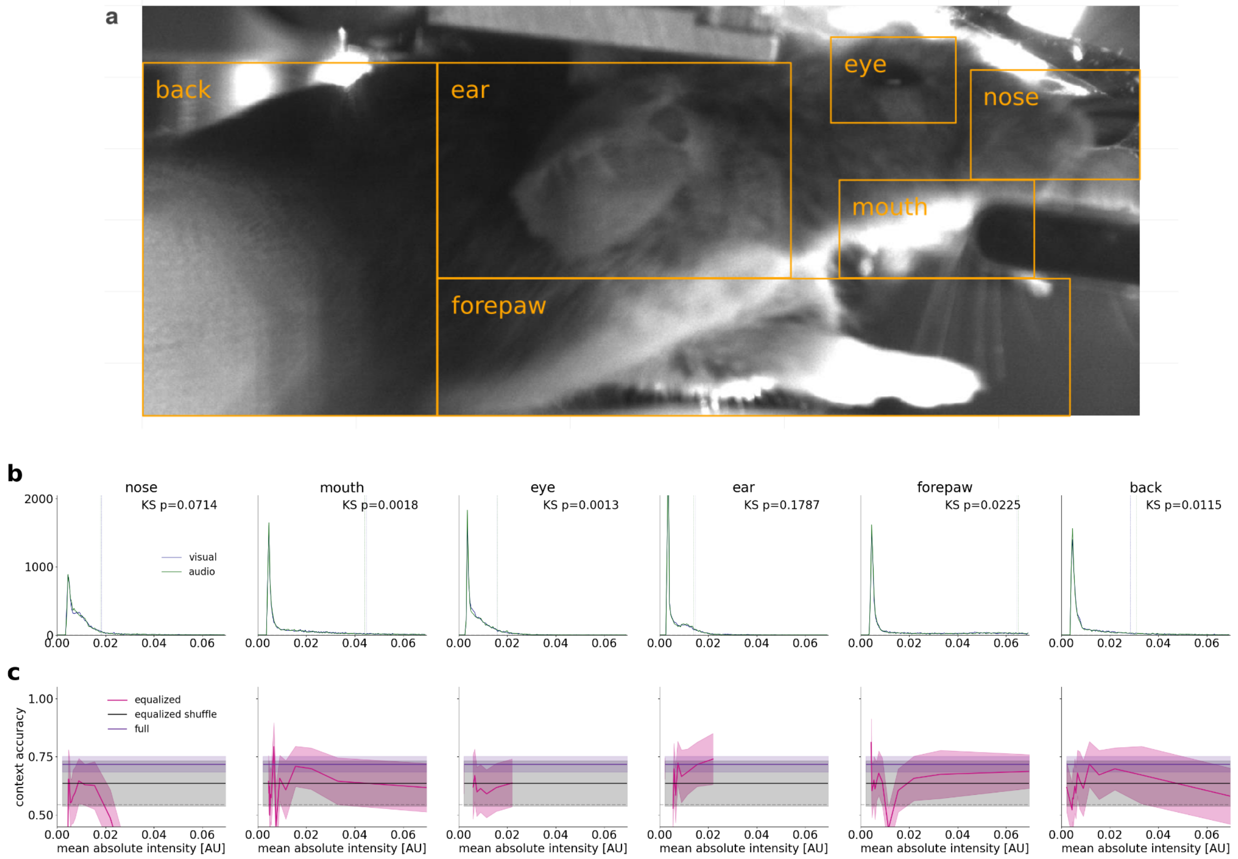

Supplementary Figure 8. Controlling context representation for body parts. a, Regions of interest around body parts. **b**, Histogram of mean differential absolute intensity levels within regions of interest in differential frames from visual (*blue*) and audio (*green*) trials. Vertical lines show 90 percentiles for each context. Small (< 0.05, < 0.01 etc. various confidence levels) Kolmogorov-Smirnov test p-values indicate difference between the two contexts. **c**, Context decoder accuracy means over the time course of trials from context-equalized number of trials at each time point at various absolute motion level intervals ('equalized', *magenta*), same as equalized, but trials randomized from all motion levels, and an average shown as a constant ('equalized shuffle', *black*), same as equalized shuffle, but from all trials ('full', *purple*). Cross-validation mean (*line*), and s.e.m (*band*). Horizontal dashed grey line shows shuffled chance level boundary for the animal.
